# Detection, Quantification, and Microlocalisation of Targets of Pesticides Using Microchannel Plate Autoradiographic Imagers

**DOI:** 10.3390/molecules16108535

**Published:** 2011-10-11

**Authors:** Mabruka H. Tarhoni, Vasanthy Vigneswara, Marie Smith, Susan Anderson, Peter Wigmore, John E. Lees, David E. Ray, Wayne G. Carter

**Affiliations:** 1 School of Biomedical Sciences, University of Nottingham, Queen’s Medical Centre, Nottingham, Nottinghamshire NG7 2UH, UK; Email: m.tarhoni@nottingham.ac.uk (M.H.T.); v.vigneswara@nottingham.ac.uk (V.V.); peter.wigmore@nottingham.ac.uk (P.W.); david.ray@nottingham.ac.uk (D.E.R.); 2 School of Graduate Entry Medicine & Health, University of Nottingham Medical School, Royal Derby Hospital, Uttoxeter Road, Derby DE22 3DT, UK; Email: mzxms2@nottingham.ac.uk (M.S.); susan.anderson@nottingham.ac.uk (S.A.); 3 BioImaging Unit, Space Research Centre, Department of Physics & Astronomy, University of Leicester, Leicester, LE1 7RH, UK; Email: lee@leicester.ac.uk

**Keywords:** microchannel plate detector, molecular imaging, pesticides, neurotoxicity, immunotoxicity, proteomics, post-translational modifications, exposure biomarkers

## Abstract

Organophosphorus (OP) compounds are a diverse chemical group that includes nerve agents and pesticides. They share a common chemical signature that facilitates their binding and adduction of acetylcholinesterase (AChE) within nerve synapses to induce cholinergic toxicity. However, this group diversity results in non-uniform binding and inactivation of other secondary protein targets, some of which may be adducted and protein activity influenced, even when only a relatively minor portion of tissue AChE is inhibited. The determination of individual OP protein binding targets has been hampered by the sensitivity of methods of detection and quantification of protein-pesticide adducts. We have overcome this limitation by the employment of a microchannel plate (MCP) autoradiographic detector to monitor a radiolabelled OP tracer compound. We preincubated rat thymus tissue *in vitro* with the OP pesticides, azamethiphos-oxon, chlorfenvinphos-oxon, chlorpyrifos-oxon, diazinon-oxon, and malaoxon, and then subsequently radiolabelled the free OP binding sites remaining with ^3^H-diisopropylfluorophosphate (^3^H-DFP). Proteins adducted by OP pesticides were detected as a reduction in ^3^H-DFP radiolabelling after protein separation by one dimensional polyacrylamide gel electrophoresis and quantitative digital autoradiography using the MCP imager. Thymus tissue proteins of molecular weights ~28 kDa, 59 kDa, 66 kDa, and 82 kDa displayed responsiveness to adduction by this panel of pesticides. The 59 kDa protein target (previously putatively identified as carboxylesterase I) was only significantly adducted by chlorfenvinphos-oxon (p < 0.001), chlorpyrifos-oxon (p < 0.0001), and diazinon-oxon (p < 0.01), the 66 kDa protein target (previously identified as serum albumin) similarly only adducted by the same three pesticides (p < 0.0001), (p < 0.001), and (p < 0.01), and the 82 kDa protein target (previously identified as acyl peptide hydrolase) only adducted by chlorpyrifos-oxon (p < 0.0001) and diazinon-oxon (p < 0.001), when the average values of tissue AChE inhibition were 30%, 35%, and 32% respectively. The ~28 kDa protein target was shown to be heterogeneous in nature and was resolved to reveal nineteen ^3^H-DFP radiolabelled protein spots by two dimensional polyacrylamide gel electrophoresis and MCP autoradiography. Some of these ^3^H-DFP proteins spots were responsive to adduction by preincubation with chlorfenvinphos-oxon. In addition, we exploited the useful spatial resolution of the MCP imager (~70 μm) to determine pesticide micolocalisation *in vivo*, after animal dosing and autoradiography of brain tissue sections. Collectively, MCP autoradiographic imaging provided a means to detect targets of OP pesticides, quantify their sensitivity of adduction relative to tissue AChE inhibition, and highlighted that these common pesticides exhibit specific binding character to protein targets, and therefore their toxicity will need to be evaluated on an individual compound basis. In addition, MCP autoradiography afforded a useful method of visualisation of the localisation of a small radiolabelled tracer within brain tissue.

## 1. Introduction

Nobel laureate Georg Von Hevesy (Nobel Prize for Chemistry, 1943) pioneered the use of radiochemicals as tracer molecules in the 1920s. Subsequently, radiochemicals have provided a means to examine countless biological processes, including the dissection of cellular signalling pathways. 

The most commonly encountered laboratory radiochemicals are alpha, beta, or gamma particles, of which beta particles are primarily utilised for analysis of biological systems. Beta-particles possess a mass and charge equal in magnitude to an electron, but differences in relative energetic strength are conferred by different laboratory radiochemicals. The commonly employed β-emitters: ^3^H, ^14^C, and ^35^S are all relatively weak β-emitters with maximum energy of 0.019, 0.156, and 0.167 mega electron volts (MeV) respectively [1,2]. Medium strength (^33^P, 0.249 MeV) and relatively strong (^32^P, 1.709 MeV) β-emitters also provide useful laboratory reagents, the latter of which has been particularly utilised for analysis of post-translational modification of proteins as phosphorylation; via the transfer of phosphate from commercial [γ-^32^P]ATP (for example refer to [3]). Use of the relatively high energetic strength ^32^P does have the requirement of containment using 1 cm of perspex to provide suitable user shielding. In addition, the half-life of ^32^P (14.3 days) may also provide time-constraints to the user to exploit the radiochemicals highest specific activity, whereas ^3^H and ^14^C β-emitters possess half-lives of 12.4 and 5,730 years, respectively. Furthermore, energy from relatively strong β-emitters is wasted by direct passage through film, although introduction of intensifying screens will limit β-particle loss and improve sensitivity, but at the expense of a reduction in signal resolution [1,2]. 

The conversion of energy from weak β-emitters to a film signal as direct autoradiography may produce signal transfer of excellent resolution, but with poor signal sensitivity. This sensitivity may be improved by the incorporation of fluors (scintillants), in which the energy of β-particles are converted to photons of blue or ultraviolet light, with the light generated captured on suitable film. However, β-emitters have the drawback of production of a random emission pathlength and self-absorption of energy from their emitting source; with the thicker the sample the greater the degree of absorption. This problem is prevalent in signal detection of β-particles incorporated into proteins that are resolved by gel electrophoresis; although this can be partly circumvented by impregnation of the gel source with a fluor, and then gel drying to reduce thickness and concentrate the signal [1,2,4]. In addition, scintillants can also be exploited for the quantitative determination of radiolabel incorporation via liquid scintillation counting.

Harnessing the energy of the β-particle (electron) as a photon may increase the sensitivity of detection by as much as a 1,000-fold [1,2]. The incident photon energy required for conversion of sufficient silver halide ions to silver atoms to produce an autoradiographic image only has a finite half-life. This incident energy half-life of a photon can be extended by incubation at low temperatures, and this will increase the likelihood of coincidence of additional incident energy photons to generate a film signal [4]. Hence signal sensitivity is facilitated by both an incorporation of a fluor and an extended autoradiographic image exposure at low temperatures, typically incubation at −80 °C. Furthermore, the threshold of signal sensitivity can be lowered, and an increase in the ability to produce a near linear correlation of incident light and film signal (facilitating quantitative measurements) can be achieved by pre-flashing the film before undertaking autoradiography [4].

However, there remains the inconvenience of relatively long autoradiographic film exposures (up to several weeks) at −80 °C for weak β-emitters such as tritium, and loss of signal resolution for relatively strong β-emitters. Therefore a market existed for suitable radioactivity detection systems that are able to provide high sensitivity for visualisation and quantitation of radiochemical incorporation across the broad range of energy strengths of β-emitters. Commercial digital autoradiography systems able to fulfil these requirements include phosphor imagers and microchannel plate (MCP) detector systems [5], and the utilisation of the latter for analysis of protein modification by pesticides will be detailed in this manuscript.

Low background noise microchannel plates provide the proximity sensor for β-particles in MCP imagers [6]. These devices were developed independently at a number of sites, including the University of Leicester Space Research Centre, at which MCP imagers were produced in order to provide photon counting in X-ray astronomy. Microchannel plate imagers are able to produce images in real-time, and linearly over six orders of signal magnitude from a detection threshold of 6 decays per minute (dpm)/mm^2^, and an intrinsic background noise of ~5 × 10^−6^ counts/second per pixel measurement [5,6,7]. They are therefore useful for detection of both relatively strong β-emitters, for example ^32^P, and also relatively weak β-emitters such as ^3^H, from which signal visualisation may still be generated within a user-friendly imaging time-course of several hours at room temperature [5,6,7]. More comprehensive details of the design and hardware associated with these MCP imagers are published elsewhere [5,6,7], and are beyond the scope of this manuscript.

The study of protein post-translational modification in biological systems may require high sensitivity, since visualisation may be limited by the protein level, and the stoichiometry of modification. The majority of proteins are likely to be modified post-translationally either enzymatically or non-enzymatically. In order to gain an insight into an alteration of biological function that may arise from a post-translational modification, there is a requirement of both tracking and quantifying the level of protein post-translational modification. However, visible protein detection is routinely limited to ~1 ng for silver stained proteins (picomoles of protein) and ~10 ng when using Coomassie staining. Radiolabel incorporation into proteins, even with weak β-emitters may provide as much as three orders of magnitude greater sensitivity than that of visual protein staining. Hence we have utilised this greater sensitivity of radiolabelling to enable detection of protein modifications that may lie below the threshold of visible protein detection methods; including those associated with protein modification by pesticides. 

There remains concerns as to the impact upon human health of environmental chemicals, including those utilised in our domestic and commercial farming procedures. Evidence from laboratory animal studies, and epidemiological analyses have suggested health concerns that may arise from acute or cumulative exposures to pesticides [8,9,10,11], and there remains the potential that health detriment may arise in part from protein-pesticide adductions. 

The primary adduction target of organophosphorus (OP) pesticides is acetylcholinesterase (AChE), a serine hydrolase resident within synapses of the central and peripheral nervous system [12]. Adduction and inactivation of AChE from pesticide binding may trigger cholinergic toxicity, and ultimately death. The serine hydrolase family of proteins, that includes AChE, is one of the largest enzyme families, estimated at approximately 1% of the eukaryotic proteome [12]. Serine hydrolase family members are structurally diverse but the majority of serine hydrolases that act on metabolites employ a serine-histidine-aspartic acid catalytic triad [12], in which the active site serine residue is a nucleophile that is susceptible to chemical adduction. Hence there is the potential of promiscuous OP binding to other serine hydrolases [13], and cross-reactivity with proteins displaying a similar chemical signature. This requires that individual OP binding site structure-activity relationships are established and their contribution to potential ill-health evaluated since their toxicokinetics may not align with those of AChE. Indeed, this inadvertent OP binding to other secondary protein targets may lie below the suggested threshold of a reduction of greater than 30% AChE inhibition to detect cholinergic toxicity [14]. These secondary OP targets may also provide useful long-lasting adjuncts to cholinesterase as biomonitors of OP exposures [15], and may have therapeutic utilisation *in vivo* as decoy surrogates (bioscavengers) that limit direct binding and toxicity attributed to AChE inhibition [16,17,18]. 

In addition, OPs and carbamates have been used to provide targeted AChE inhibition in the treatment of the neuromuscular disease myasthenia gravis, and neurodegenerative diseases such as Alzheimer’s disease, but side-effects of these anti-cholinesterase pharmacological treatments have been reported [19]. It will therefore be of interest to define any additional protein secondary targets and allay fears of additional drug-binding side-effects from cholinomimetics.

The study of low-dose pesticide exposures is facilitated by the provision of sensitive methods of detection of protein adduction, and this has been achieved by the incorporation of radiolabels into proteins and radiolabel signal visualisation by autoradiography using MCP imaging. We have investigated thymus tissue protein binding targets of OP pesticides. We have focussed our studies upon OP pesticides that are in common use within the UK: azamethiphos, chlorfenvinphos, malathion, diazinon, pirimiphos-methyl, and also chlorpyrifos, widely used within the USA. Many pesticides are utilised as inactive precursor thions, but become bioactivated *in vivo* to their active oxon counterparts. Hence our strategy for analysis of pesticide targets within tissues has involved either direct tissue incubation with the active pesticide-oxon *in vitro*, or dosing of animals *in vivo*, with pesticide at a concentration and time known to produce a desired level of tissue AChE inhibition. After pesticide incubation, proteins were radiolabelled with the broad and stable serine hydrolase inhibitor ^3^H-diisopropylfluorophosphate (^3^H-DFP). If a pesticide had bound active site serine residues, or a similar chemically reactive group, then a reduction in subsequent ^3^H-DFP radiolabelling was used as a means to locate the pesticide targets. In addition, if radiolabelled ligands are available, we show that the MCP imager provides a means to detect and microlocalise pesticide within tissue sections.

## 2. Results and Discussion

**Figure 1:**
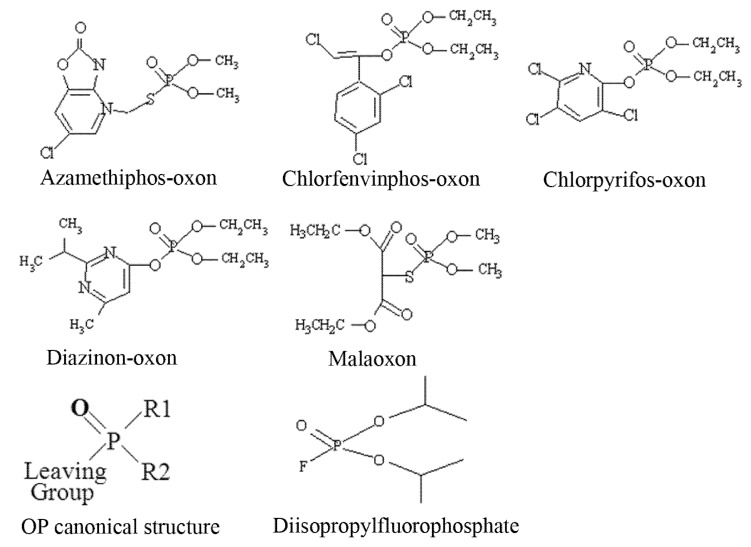
Structures of pesticides employed for *in vitro* analyses.

Our strategy to detect active OP pesticide targets has involved either preincubation of tissue with pesticide *in vitro*, or dosing of animals *in vivo*, and then subsequently radiolabelling remaining free OP binding sites with ^3^H-DFP. Organophosphorus pesticides have a canonical structure comprised of a central phosphorus atom bound to two R groups, a leaving group, and double bonded to either a sulphur (thion) or oxygen (oxon). Thions may undergo bioactivation *in vivo* to their oxon counterparts, and hence for *in vitro* analyses pesticide oxons were used directly. The structures of the pesticides used for these *in vitro* analyses as their active oxon forms are listed as Figure 1. 

The OP pesticide canonical structure and that of the radiolabelling agent DFP are also included. Pesticide binding to tissue protein targets was evident as a reduction in radiolabel incorporation relative to control (solvent) incubations. We have focussed our recent studies on an examination of the pesticide targets within brain tissue, immune system tissues, and blood [9,15,20]. We have expanded our studies to include the immune system, since it requires the activity of a number of serine hydrolase family members, for example, proteins involved in the complement cascade, to function optimally. Herein we report details of studies undertaken with thymus tissue which is a component of primary lymphatic tissue, and functions to produce immunocompetent T-lymphocytes. 

**Figure 2:**
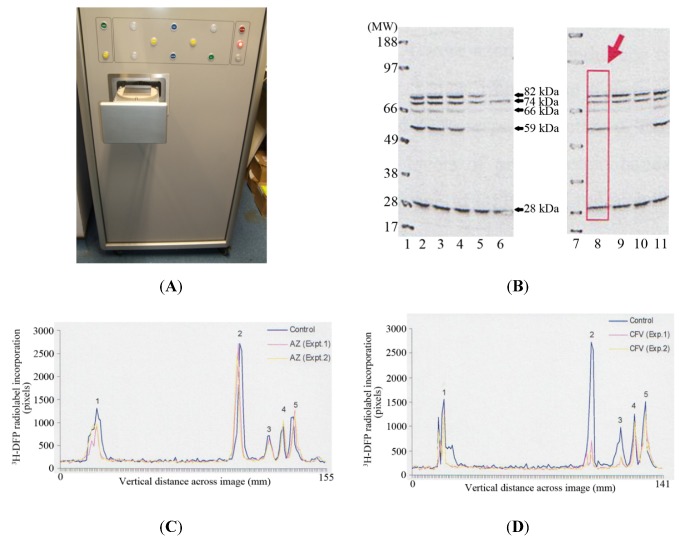
Detection and quantification of pesticide targets *in vitro* within rat thymus tissue using MCP autoradiography. (**A**) Photographic image of the front of the MCP imager with the loading drawer open to reveal the loading plate onto which a PVDF membrane or slides are applied. **(B)** An example of 1D PAGE and autoradiography. Gel lanes 1 and 7 are positions of the molecular weight markers. Gel lanes 2 and 8 are control thymus tissue protein radiolabelling levels. Gel lanes 3 and 4 are preincubations with azamethiphos-oxon at 35% and 89% tissue AChE inhibition respectively. Gel lanes 5 and 6 are preincubations with chlorpyrifos-oxon at tissue AChE inhibitions of 38% and 98% respectively. Gel lanes 9 and 10 are preincubations with chlorfenvinphos-oxon at tissue AChE inhibitions of 30 and 63% respectively. Gel lane 11 is a preincubation with malaoxon, at 70% tissue AChE inhibition. **(C)** Examples of the quantitation of radiolabel incorporated into protein targets 1–5 (^3^H-DFP proteins of ~28, 59, 66, 74, and 82 kDa respectively) after preincubation with relatively low doses (≤30% tissue AChE inhibition) of azamethiphos-oxon. **(D)** Examples of the quantitation of radiolabel incorporated into protein targets 1–5 after preincubation with relatively low doses (≤30% tissue AChE inhibition) of chlorfenvinphos-oxon. For plotting, the background autoradiographic intensity at each pixel measurement within a gel lane was subtracted from the test level recorded. The results shown are representative of at least 10 independent experiments with each of the pesticides.

The MCP imager has dimensions of 150 cm (height) × 69 cm (width) × 93 cm, with a loading drawer which can be triggered to open to reveal a loading plate of 9.3 cm × 9.3 cm (Figure 2A). A PVDF membrane or slides are applied to the loading plate, and then the loading drawer triggered to close, after which the PVDF membrane or slides are applied to the microchannel plates for autoradiographic imaging. This autoradiographic imaging window is therefore ideally suited to visualise protein radiolabelling after protein separation by 1D PAGE using standard mini-gels which have typical dimensions of 7 cm (length) × 8 cm (width). 

Thymus proteins were preincubated *in vitro* with solvent or pesticide for 20 minutes at room temperature. A proportion of incubate was removed and the level of tissue AChE inhibition measured. The remaining incubate was radiolabelled with ^3^H-DFP, and proteins resolved by 1D PAGE, and then transferred to a PVDF membrane for autoradiography. Tritiated-DFP radiolabelled proteins were characterised by their molecular weight, and have been marked with arrows at ~28 kDa, 59 kDa, 66 kDa, 74 kDa, and 82 kDa in Figure 2B. Pesticide adducted proteins were recognised by a reduction in radiolabelling from those of control (solvent) incubations. Thus for example, preincubation of tissue with chlorpyrifos-oxon and production of 38% tissue AChE inhibition, resulted in adduction of the 59 kDa, 66 kDa, and 82 kDa proteins, and hence a reduction in their subsequent ^3^H-DFP radiolabelling—refer to Figure 2B, Lane 5. From an array of similar experiments we have been able to characterise the major thymus tissue protein targets and moreover, qualitatively determine if these proteins were sensitive to pesticide binding at relatively low (≤30% AChE inhibition) or relatively high tissue tissue AChE inhibitions.

In addition to provision of rapid real-time autoradiographic visualisation of radiolabel binding, the MCP imaging device also possesses intrinsic software (Quant scan) capable of quantitation of the levels of autoradiographic signal in pixels. Numerical values for the autoradiographic intensity were measured vertically down each of the gel lanes (such as that marked with a rectangular box and large arrow in lane 8 of Figure 2B), and the signal plotted in millimetres across the gel image using Excel—Figure 2C, Figure 2D. The dark outer solid line in each of these images represents the level of ^3^H-DFP radiolabel incorporated into each of the protein bands detected in control incubations. The ~28 kDa protein band is labelled as protein target 1, the 59 kDa band protein target 2, 66 kDa band protein target 3, 74 kDa is protein target 4, and 82 kDa protein target 5.

This quantitative determination of autoradiographic signal intensity provided a measure of the relative levels of radioactivity incorporated into each protein target, but moreover, provided a means to quantify the fall in radioactivity that arose after preincubation with each of the pesticides. Thus, for example, azamethiphos-oxon in two separate experiments had no significant effect on the levels of ^3^H-DFP radiolabelling of protein targets 2–5, but did induce a slight reduction in the radioactivity incorporated into protein target 1 (~28 kDa)—refer to Figure 2C. Chlorfenvinphos-oxon in the two experiments shown also produced a reduction in the radiolabelling of the ~28 kDa protein, and significant reduction in radiolabelling of proteins targets 2 and 3 (59 kDa and 66 kDa respectively)—refer to Figure 2D. None of the pesticides assessed exhibited significant adduction of the 74 kDa protein (protein target 4) at relatively low doses (≤ 30%) of tissue AChE inhibition—refer to Figure 2B–D.

**Figure 3:**
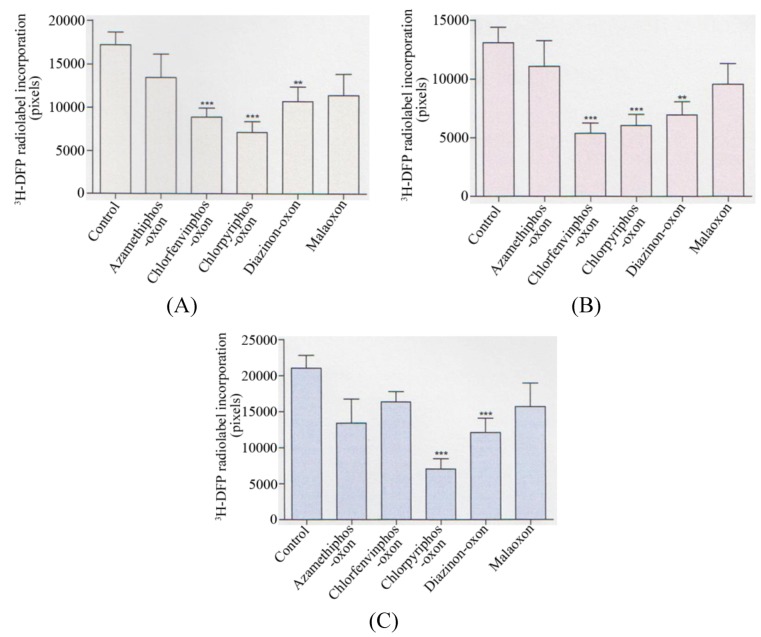
Summary of quantification of *in vitro* pesticide binding to protein targets in thymus tissue.

Collectively, when analysed statistically, there was a significant reduction in the radiolabelling of the 59 kDa protein target (p < 0.001) with chlorfenvinphos-oxon, chlorpyrifos-oxon (p < 0.0001), and diazinon-oxon (p < 0.01) when average values of AChE inhibitions of 30%, 35%, and 32% were used respectively–refer to Figure 3A. At these average AChE inhibitions there was also a significant reduction in the 66 kDa protein target by the same three pesticides (p < 0.0001), (p < 0.001), and (p < 0.01) respectively–refer to Figure 3B. For the 82 kDa protein target, only a significant reduction in radiolabelling was seen with chlorpyrifos-oxon (p < 0.0001) and diazinon-oxon (p < 0.001)–refer to Figure 3C.

Quantification of the reduction in ^3^H-DFP radiolabelling of thymus tissue protein targets after a preincubation with pesticide to produce 30–35% tissue AChE inhibition. **(A)** 59 kDa protein target, **(B)** 66 kDa protein target, **(C)** 82 kDa protein target. Figures are representative of at least 10 independent experiments with each of the pesticides. Figures display mean changes ±SEM. Asterisks denote significant changes from control incubations.

That a fall in ^3^H-DFP radiolabelling of a protein target directly correlated with OP binding, was previously validated by a demonstration of reduced radiolabelling of recombinant AChE with increased OP preincubation, and corresponding increased enzymatic inhibition [20]. This methodology has highlighted that these commonly employed pesticides do not exhibit identical (generic) binding characteristics to protein targets, and therefore their structure-activity relationships will need to be evaluated on an individual compound basis. 

The thymus proteins radiolabelled with ^3^H-DFP and resolved by 1D PAGE did not perfectly overlay with proteins visualised by either Coomassie or silver staining (results not included), consistent with autoradiography sensitivity above that achieved with protein staining. Autoradiographic images were captured after a 24 hour exposure exploiting the MCP imager’s detection threshold of 6 dpm/mm^2^, and it would seem unlikely, unless for relatively long exposures, that conventional film autoradiography would have been sensitive enough to have enabled us to detect these pesticide targets by a quantifiable fall in ^3^H-DFP autoradiography after pesticide preincubations. By comparison, when using pre-exposed film a detection threshold for ^3^H of 300 dpm for a gel band of 1 cm × 1 mm has been suggested [4]. Similarly, a minimum radiolabelling of 20,000 dpm/cm^2^ for autoradiography and 500 dpm/cm^2^ for fluorography have been reported for analyses using nitrocellulose filters [21].

One dimensional PAGE may suffer from the limitation of insufficiently resolving proteins of similar molecular weight, and/or the masking of pesticide responsive protein targets by the co-localisation of ^3^H-DFP radiolabelled proteins that were unresponsive to pesticides. The protein band labelled with ^3^H-DFP that resolved to a molecular weight of ~28 kDa (target 1) was an example of a dense protein band comprised of more than one radiolabelled protein, and/or presumed post-translational modifications of the same protein. Protein band heterogeneity hindered quantitation of the level of pesticide adduction of this protein target. Hence protein targets for which heterogeneity is suspected may be better resolved and adduction examined after protein separation by two dimensional techniques. The MCP analysis window is also of suitable size to facilitate protein separation by 2D-PAGE, since commercially available isoelectric focussing strips of 7 cm in length can be coupled to mini-gel protein separation [22,23].

**Figure 4:**
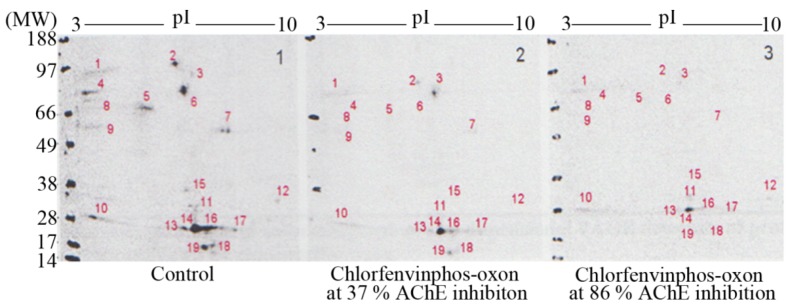
Characterisation of thymus tissue chlorfenvinphos-oxon pesticide targets *in vitro* after protein resolution by 2D-PAGE.

Resolution of ^3^H-DFP radiolabelled thymus proteins by 2D-PAGE and 48 hour MCP autoradiography revealed up to nineteen ^3^H-DFP radiolabelled protein spots, some of which were just above detection threshold–refer to Figure 4. The strategy of preincubation with pesticides prior to ^3^H-DFP radiolabelling was also employed to detect which protein spots were sensitive to pesticide binding–Figure 4. The protein spots resolved by 2D-PAGE and assigned to the 59 kDa, 66 kDa, and 82kDa protein bands displayed identical selective pesticide responsiveness as that characterised using 1D PAGE, but only some of the radiolabelled proteins of molecular weight 26-29 kDa were responsive to pesticide adductions.

Thymus proteins were preincubated *in vitro* with chlorfenvinphos-oxon, the level of tissue AChE measured, and then proteins radiolabelled with ^3^H-DFP, resolved by 2D-PAGE, and radiolabelled proteins visualised by MCP autoradiography. Protein target adduction was monitored by a reduction in ^3^H-DFP radiolabelling. Up to 19 ^3^H-DFP radiolabelled spots were visualised after 2D-PAGE autoradiography (left panel). Some of the resolved proteins were sensitive to adduction after extract preincubation with chlorfenvinphos-oxon at relatively low AChE inhibition (middle panel), and more so after extract preincubation with chlorfenvinphos-oxon at relatively high AChE inhibition (right panel). Image is representative of three independent experiments with chlorfenvinphos-oxon at relatively low and high tissue AChE inhibitions.

In addition to resolution of heterogeneous protein bands, 2D-PAGE also provides isoelectric point (pI) information, and hence the employment of bioinformatic search engines. The combination of selection character of a hydrolase, with specified molecular weight and pI enabled engagement of the TagIdent bioinformatic search engine tool available at the Expasy website (www.expasy.org), to provide a list of candidate protein targets. Thus for example, a hydrolase of approximate molecular weight 82 kDa (±1%), displaying a pI of ~5.3 (range 5.1–5.5) (protein spot denoted number 6 in Figure 4), yields a list of only four potential protein candidates: calpain-1 catalytic subunit, acylamino-acid releasing enzyme (previously known as acyl peptide hydrolase), isoform PDE4B3 of cAMP-specific 3,5’-cyclic phosphodiesterase, and membrane mettalo-endopeptidase-like 1. 

This 82 kDa protein target, and likewise those of 66 kDa, 59 kDa, and the heterogeneous ~28 kDa protein targets were all at relatively low levels in the thymus tissue preparation, and hence direct mass spectrometric identification of protein bands resolved by 2D-PAGE was not possible. However, even with low abundance proteins such as these, the ability to track their presence via ^3^H-DFP binding enabled us to employ column chromatographic methods to enrich the pesticide targets to a sufficient level for protein identification by mass spectrometry, and the 82 kDa protein target was identified as acylamino-acid releasing enzyme, in support of the bioinformatic approach [15,20,24].

Purification of the 66 kDa protein target led to its identification as serum albumin [9]. Albumin is not a serine hydrolase but is bound and/or adducted by a number of pesticides, since it possesses a chemically reactive tyrosine similar to that of the active site serine nucleophile present in serine hydrolases [9,15,20,25,26,27,28]. It was therefore not detected using the bioinformatic search for candidate serine hydrolases.

We have also purified the 59 kDa pesticide target to near homogeneity, and undertook characterisation of the enzyme’s physical and chemical properties [15]. In agreement with other independent laboratories we suggest that the 59 kDa pesticide target is carboxylesterase I, displaying expression in immune cells and immune tissues [25,29,30,31]. Current experiments are underway to purify the pesticide responsive proteins within the broad ~28 kDa protein band to a sufficient level to enable identification by mass spectrometry.

Our strategy for detection of OP pesticide protein binding as a reduction in subsequent ^3^H-DFP radiolabelling is not without drawbacks, and may miss some pesticide binding targets. There may be proteins that are only sensitive to a specific pesticide and not ^3^H-DFP, or proteins that possess only a few OP sensitive target amino acids and these become saturated with pesticide binding and thereby limit any significant subsequent ^3^H-DFP binding. However, in the absence of commercially available pesticide radioligands this method has proved a useful approach, and has resulted in the detection of hitherto unknown *in vivo* pesticide targets that overlap with fluorescent ligand binding approaches adopted by other laboratories [9,15,20,24,25,32]. Indeed, our competitive strategy of preincubation with a pesticide *in vivo* to deplete reactive binding sites has also been recently employed using a fluorophosphonate probe [32].

The utilisation of MCP autoradiographic imaging after protein separation by 1D or 2D-PAGE is applicable to the analysis of other post-translational modifications that may require high sensitivity of radiolabel detection. If the post-translationally modified proteins of interest are sufficiently abundant to be visualised by Coomassie or silver staining techniques, the MCP autoradiographic image can be utilised to provide a template to overlay directly with the resolved proteins. We adopted this proteomic strategy to detect and identify proteins modified post-translationally as carboxyl methylation by detecting methylated proteins using exogenous methyltransferase and radiolabelled methyl donor [33]. Additionally, application of MCP imaging to other radiolabelled biological assays that require sensitive, quantifiable measurements of electron emission such as lymphocyte proliferation assays are feasible [34].

**Figure 5:**
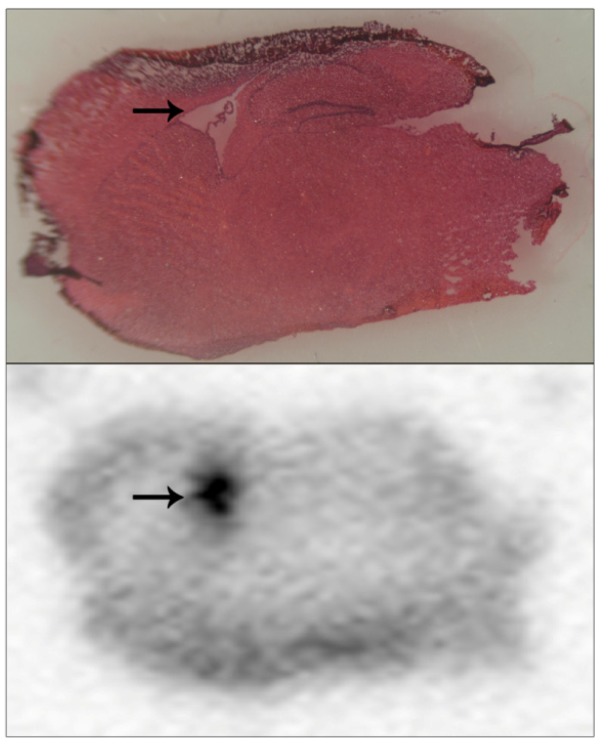
Pesticide radiolabel microlocalisation within brain tissue slices.

Our strategy of competitive binding of pesticide to a protein target prior to radiolabelling with ^3^H-DFP is not required if pesticides or other small molecules are available as radiolabelled ligands. Currently, radiolabelled pesticides are not commercially available, but some may be produced as a custom synthesis. Since the MCP autoradiographic imager displays useful spatial resolution (~70 μm) [5], we exploited this to examine microlocalisation *in vivo* for a custom synthesis radioligand. We prepared brain sections from animals 24 hours after they had been dosed with radiolabelled pesticide, and used the sensitive MCP autoradiographic imaging to provide insight into radiolabel microlocalisation within brain tissue. A prominent hot-spot of radiolabel was evident within the same tissue sections of each animal analysed, an example of which is included as Figure 5 (lower panel, hot-spot indicated with an arrow). These tissue sections were also examined using light microscopy of tissue slices that had been stained with H&E in order to localise radiolabel accumulation to brain anatomical regions. Even after freeze-drying and subsequent H&E staining, brain structural landmarks were preserved within tissue sections. The hot-spot of radiolabel incorporation overlaid with the brain lateral ventricles, which are flattened and appear as a white region on the slide due to the freeze-drying process—Figure 5 (upper panel, lateral ventricles indicated with an arrow). Presumably, accumulation of radiolabelled pesticide within the lateral ventricles reflected equilibrium with the cerebrospinal fluid within them. Figure 5 is included to illustrate the usage of this methodology for localising radiolabel within tissue sections, and more extensive details regarding these specific pesticide accumulations will be published elsewhere. Certainly, we would foresee that this approach of how to detect and microlocalise small radiolabelled molecules within tissue sections would have general methodology application.

Brain tissue from mice dosed with a radiolabelled pesticide was cryosectioned and brain tissue slices applied to a standard slide, freeze dried, and subjected to 24 hours of MCP autoradiography (lower panel). Tissue sections were H&E stained (upper panel), and light microscopy used to overlay brain regional structure with radiolabel accumulations. A hot-spot of radiolabel accumulation was localised to the lateral ventricles, and has been indicated with arrows.

## 3. Experimental

### 3.1. Materials

The organophosphorus pesticides azamethiphos-oxon (*S*-6-chloro-2,3-dihydro-2-oxo-1,3-oxazolo-[4,5-*b*]pyridin-3-ylmethyl *O*,*O*-dimethyl phosphorothioate), chlorfenvinphos-oxon (2-chloro-1-(2,4-dichlorophenyl)vinyl diethyl phosphate), and malathion (diethyl(dimethoxyphosphino-thioylthio)succinate) and its corresponding oxon (malaoxon) were all purchased from QMX Laboratories Ltd., Thaxted, UK, and were of 95–99.5% purity. Chlorpyrifos (*O*,*O*-diethyl-*O*-(3,5,6-trichloro-2-pyridyl)phosphorothioate) and diazinon (*O*,*O*-diethyl *O*-2-isopropyl-6-methylpyrimidin-4-yl phosphorothioate) as their corresponding oxons were purchased from Greyhound Laboratories, Birkenhead, UK, at 97.2–99.4% purity. The structures of the pesticides used for *in vitro* analyses are shown in Figure 1. For *in vitro* assays, pesticides were prepared as 100 mM stock solutions in ethanol (Sigma, HPLC grade, <0.10% water), except azamethiphos-oxon which was at 50 mM, and were stored at 4 °C for up to 2 weeks. Pesticides were diluted in phosphate buffered saline (PBS) to required concentrations just prior to use. Tritiated-diisopropylfluorophosphate (^3^H-DFP) at a specific activity of 150 GBq/mmol was purchased from Perkin Elmer, Boston, USA. NuPAGE Novex pre-cast gels (4–12% Bis-Tris gels for one-dimensional (1D) polyacrylamide gel electrophoresis (PAGE) and 4–12% Bis-Tris Zoom gels for two dimensional (2D) PAGE, 3-(N-morpholino)propanesulfonic acid (MOPS)-SDS running buffer, and all other PAGE reagents were purchased from Invitrogen.

### 3.2. Tissue Preparations

Male C57BL/6J mice or male F344 strain rats were used for experiments. Animals were housed, and dosed according to previous publications [9,15,20]. All animal procedures were approved by the University of Nottingham Local Ethical Review Committee and were carried out in accordance with the Animals Scientific Procedures Act (UK) 1986. Thymus tissue preparations were as detailed previously [9,15,20], with protein concentrations in homogenates measured using the DC Protein assay (Biorad) using bovine serum albumin as a protein standard. For microlocalisation studies, mice brains were divided into equal halves, immediately snap-frozen in liquid isopentane at −20 °C, and then cryosectioned using a Leica CM3050 cryostat in the para-sagittal direction with step serial sections of 20 μm thickness taken in the midline plane. One section of every five was retained (generating a total of ~50 sections/hemi-brain), and three sections mounted per gelatin-coated slide. Slide-mounted tissue sections were freeze dried to fix the tissue and minimise *post-mortem* signal diffusion, and then tissue sections were maintained *in vacuo* within a vacuum dessicator prior to image analysis. Brain tissue section slides were covered with a 5 μm film of polyethylene terephthalate (PETP) (Goodfellow Cambridge Ltd, UK) and then applied to the MCP imager. The PETP film was employed to limit contamination of radiolabel from tissue sections to the imager microchannel plates. Microlocalisation of radiolabelled ligand was monitored by real-time MCP autoradiography for 24 hours, with images shown typical of that produced from sections taken from a total of four similarly dosed animals. Slide-mounted freeze-dried tissue sections were stained using standard H&E staining and then viewed using an Olympus SZ-PT binocular light microscope, with digital images of brain tissue sections captured with a Canon EOS 7D camera.

### 3.3. Acetylcholinesterase Measurements

Thymus tissue AChE activity measurements were based upon the spectrophotometric method described by Ellman *et al*. [35]. Spectrophotometric measurements were performed at 412 nm in a Perkin Elmer Lambda 2S spectrophotometer operated using UV KinLab software as described previously [15].

### 3.4. Protein Separation and Autoradiography

Thymus tissue homogenates were incubated with either pesticide, or PBS as solvent for 20 minutes at room temperature. Proteins were then radiolabelled by incubation with 24 μM ^3^H-DFP (final concentration) for 1 hour at 37 °C. Proteins were then heat denatured and resolved by 1D or 2D PAGE, and proteins transferred to a polyvinylidene difluoride (PVDF) membrane for autoradiography as published previously [33,36]. Approximately 0.25 μL of 100 KBq ^14^C-amino acid mixture (1.85 MBq/mL, Amersham) was applied to PVDF blots at the positions of the molecular weight markers to provide an estimation of the molecular weight of ^3^H-DFP radiolabelled proteins after autoradiography. Blots were subjected to 24 hours of autoradiography within a MCP detector for 1D PAGE experiments, and 48 hours of autoradiography for 2D-PAGE resolved proteins. Radiolabelled protein bands in autoradiographic images were quantified using Quant scan software (beta autoradiographic image acquisition software), with band intensities (in pixels) plotted using Excel to determine relative radiolabel incorporations. Radiolabel incorporations into protein targets were analysed by one-way analysis of variance (ANOVA) with *post-hoc* test (Bonferroni’s multiple comparison test) using Prism software.

## 4. Conclusions

The study of protein post-translational modifications has been advanced by the commercial availability of radiochemical ligands. The relative ease of synthesis and a relatively long half-life, render tritium incorporation into radiochemicals a suitable tracer ligand to study many biological processes. However, tritium usage may be hindered by the limit in the detection threshold and signal linearity of conventional film autoradiography. MCP digital autoradiography displays superior detection sensitivity and over a more comprehensive signal magnitude than that of film autoradiography. These traits may be exploited for the detection and quantification of protein modifications that lie well below protein visibility levels, such as the protein modifications by pesticides detailed in this manuscript. The MCP devices also possess useful spatial resolution to enable microlocalisation of small molecule radioligands, and collectively these qualities will continue to assist with the dissection and understanding of biological systems.
